# Realizing the Continuous Chemoenzymatic Synthesis of Anilines Using an Immobilized Nitroreductase

**DOI:** 10.1021/acssuschemeng.3c01204

**Published:** 2023-06-02

**Authors:** Sebastian C. Cosgrove, Gavin J. Miller, Amin Bornadel, Beatriz Dominguez

**Affiliations:** † School of Chemical and Physical Sciences & Centre for Glycoscience, Keele University, Keele, Staffordshire ST5 5BG, United Kingdom; ‡ Johnson Matthey, 28 Cambridge Science Park, Milton Rd, Cambridge CB4 0FP, United Kingdom

**Keywords:** biocatalysis, nitroreductase, flow biocatalysis, enzyme immobilization, anilines

## Abstract

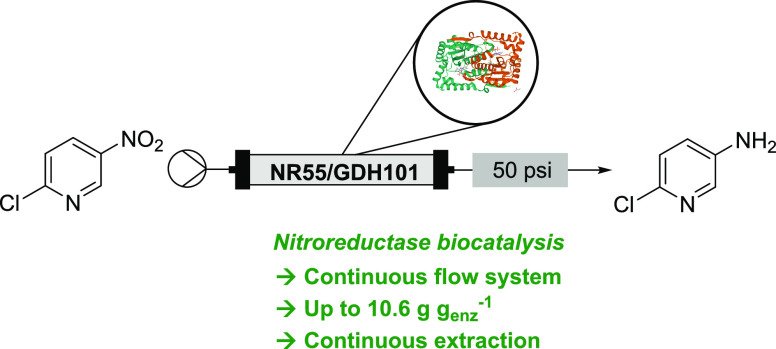

The use of biocatalysis for classically synthetic transformations has seen an increase in recent years, driven by the sustainability credentials bio-based approaches can offer the chemical industry. Despite this, the biocatalytic reduction of aromatic nitro compounds using nitroreductase biocatalysts has not received significant attention in the context of synthetic chemistry. Herein, a nitroreductase (NR-55) is demonstrated to complete aromatic nitro reduction in a continuous packed-bed reactor for the first time. Immobilization on an amino-functionalized resin with a glucose dehydrogenase (GDH-101) permits extended reuse of the immobilized system, all operating at room temperature and pressure in aqueous buffer. By transferring into flow, a continuous extraction module is incorporated, allowing the reaction and workup to be continuously undertaken in a single operation. This is extended to showcase a closed-loop aqueous phase, permitting reuse of the contained cofactors, with a productivity of >10 g_product_ g_NR-55_^–1^ and milligram isolated yields >50% for the product anilines. This facile method removes the need for high-pressure hydrogen gas and precious-metal catalysts and proceeds with high chemoselectivity in the presence of hydrogenation-labile halides. Application of this continuous biocatalytic methodology to panels of aryl nitro compounds could offer a sustainable approach to its energy and resource-intensive precious-metal-catalyzed counterpart.

## Abstract 2

Immobilization of a nitroreductase enzyme permits continuous chemoenzymatic synthesis of anilines.

## Introduction

Biocatalysis has become an integral part of the synthetic chemists’ toolbox over the last few decades.^1^ The speed with which DNA can be translated to function, coupled with the ever-increasing capability of protein engineers, means the repertoire of synthetic chemistries now available to enzymes is growing rapidly.^2^ This includes transformations once thought of as impossible for enzymes, such as cyclopropanation,^3,4^ C–N,^5^ C–Si,^6^ and C–C bond forming reactions.^7^ Biocatalysis has also seen an increase in application toward complex biomolecules including peptides,^8^ (oligo)nucleotides,^9^ and carbohydrates.^10^ An area where biotransformations hold particular promise in is the direct replacement of synthetic methods that are perceived as unsustainable, in the long term.^11^ For example, precious metal-mediated hydrogenation is an important industrial process, which has been applied to the reduction of numerous functionalities, e.g., nitro, carbonyl, alkene.^12^ It does, however, run at high pressure, use hydrogen gas and typically requires expensive and resource-intense group 10 transition metals, such as palladium.^13^

**Figure 1:**
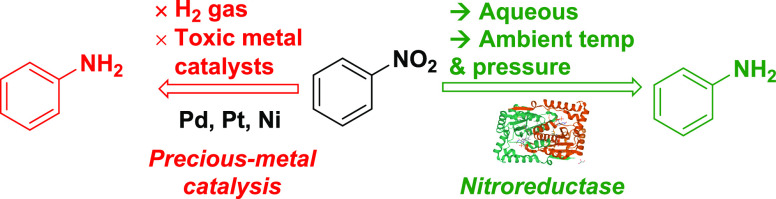
Comparison of precious metal and biocatalytic approaches to aryl nitro reduction.

A biotransformation that could replace one such hydrogenation process is the use of nitroreductase (NR, EC = 1.7.1.16) enzymes for nitroaromatic group reduction. NRs selectively mediate the reduction of aryl nitro groups. There are two classes of NR, namely, type I, which are oxygen-insensitive and type II, which are oxygen-sensitive.^14,15^ Comprehensive works have elucidated much of the mechanism of action for NRs and shown they do not convert from the hydroxylamine to the aniline, indeed stopping at the hydroxylamine via short-lived nitroso intermediates.^16,17^ They can be coupled with other catalysts to effect overall reduction to the more synthetically useful anilines, however (Figure 1).^18^ This presents a chemoenzymatic alternative to synthetic hydrogenation, running under atmospheric pressure, in aqueous media, and with perfect chemoselectivity. Several transition metal co-catalysts that can be used for hydroxylamine reduction in conjunction with NR mutants have been disclosed.^18−20^ This permits the preparation of highly functionalized anilines using relatively mild and inexpensive conditions.^17^ There is therefore an opportunity for NR enzymes to be used within bioremediation of nitroaromatic compounds, with bacteria identified that use such compounds as energy sources.^21,22^ Also, as stated, the significance of anilines in the development of bioactive molecules, coupled with the abundance of nitroaromatics as synthetic building blocks, offers new biosynthetic routes for NRs as replacements of synthetic hydrogenation catalysts in both discovery and process chemistry. Ultimately, switching to biocatalytic processes such as this to prepare aniline derivatives offers much in the way of sustainability, including reduced reliance on precious metals, lower energy requirements, and aqueous alternatives to bio-incompatible organic solvents.

Alongside protein engineering, the process in which enzymes are subsequently used has to be improved, to access optimum biocatalyst function. Synthetic bioprocess design has, therefore, an essential role to play in the wider adoption of enzymes and biocatalysis.^23^ One particular process tool that can offer numerous benefits is continuous flow, the running of reactions under a continuous regime, rather than as sequential batch operations.^24−27^ There has been a swift rise in the number of reports associated with continuous flow biocatalysis, showcasing several benefits that continuous reactors can offer to enzymatic transformations.^28,29^ A key technology that is used in conjunction with flow biocatalysis is enzyme immobilization.^26,30,31^ This can offer cost–benefit permitting reuse of the enzyme and can simplify downstream processing through removal of soluble protein from the reaction mixture. Herein, the investigation of the immobilization of a NR and its transfer into a continuous flow reactor is discussed.^20^ Immobilization of the biocatalyst is investigated in conjunction with the optimization of continuous reactions using the immobilisate, to allow better understanding of how NRs can be used more efficiently for the synthesis of aromatic aniline building blocks.^32^

## Results and Discussion

### Batch Optimization

**Scheme 1:**
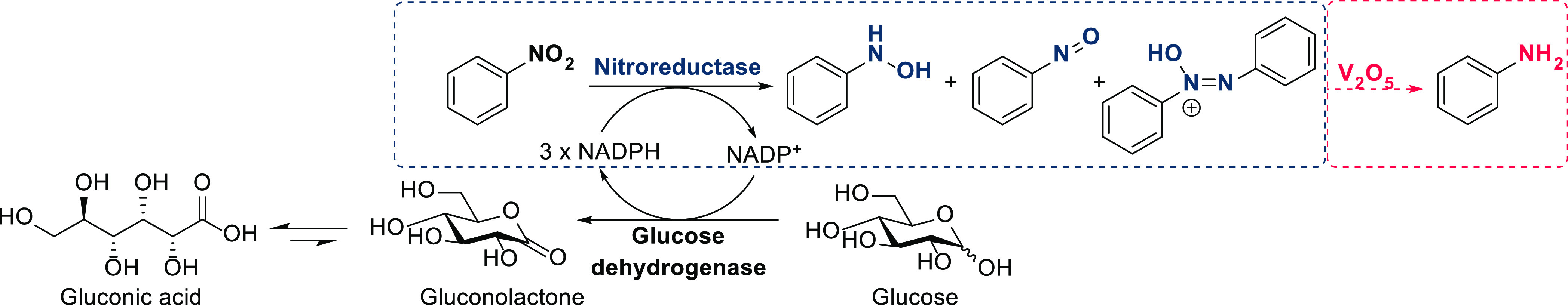
Nitro Reduction Using the Combined Nitroreductase (Blue Products)/Vanadium (Red Product) Catalysis Approach

The first step was to optimize the performance of a selected NR, with 2-chloro-5-nitropyridine **1a** chosen as the model substrate. An aqueous buffer and toluene biphase has been reported previously;^20^ however, as the intention was to run this reaction as a continuous process, it was considered simpler to adopt a homogenous solution. Several chemical co-catalysts have been used with NRs to effect full conversion to the aniline.^18^ We opted to use V_2_O_5_ due to previous success with the NR-55 chosen from the Johnson Matthey collection. An engineered glucose dehydrogenase (GDH-101) was used for NADPH recycling (Scheme 1).

An overnight reaction to reduce **1a** with 10% v/v DMSO went to full conversion (Table 1, entry 1). A time-course analysis (see ^1^H NMR data in the SI) revealed that the reaction went to completion in 2 h (entry 2). The substrate concentration could be increased up to 60 mM and still fully consume **1a**, although this resulted in formation of a small amount the azoxy dimerization product **4a** (entry 3). Despite being efficient at the 50 mM substrate concentration (entry 4), pyridine **1a** was highly insoluble above ∼7.5 mM, even with 10% DMSO, and increasing this above 20% v/v had little effect on solubility and also a negative impact on enzyme activity. Therefore, other co-solvents were tested to see if they could better solubilize **1a** and not impact enzyme performance. Acetonitrile appeared to have no impact and could be used interchangeably with DMSO (entry 5), although **1a** still did not solubilize well above ∼10 mM. Immiscible solvents EtOAc and toluene could also be used, with EtOAc proceeding to full conversion (entries 6 and 7). The final batch process could run efficiently at the 50 mM substrate concentration and reach full conversion in under 2 h, despite the fact **1a** was highly insoluble at this concentration (entry 4). During optimization, only trace amounts of **3a** were observed and very little **4a** was encountered. This is likely due to the effectiveness of V_2_O_5_ as a co-catalyst.^18,19,33^

**Table 1:**
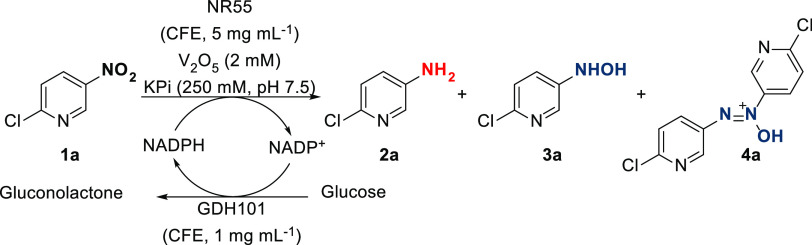
Optimization of NR-Mediated Reduction of Pyridine **1a**^a^

run	co-solvent (v/v)	substrate	time	ratio^b^**1a**:**2a**:**3a**:**4a**			
1	10% DMSO	20 mM	20 h	0	100	0	0
2	10% DMSO	20 mM	2 h	0	100	0	0
3	10% DMSO	60 mM	2 h	2	95	0	3
4	10% DMSO	50 mM	2 h	0	95	0	5
5	10% MeCN	50 mM	2 h	7	93	0	0
6	10% EtOAc	50 mM	2 h	0	98	0	2
7	10% Toluene	50 mM	2 h	22	78	0	0

a Standard conditions: substrate **1a**, NR-55 CFE (5 mg mL^–**1**^), GDH-101 CFE (1 mg mL^–**1**^), glucose (4 × [S]), NADP^+^ (1 mM), V_2_O_5_ (2 mM), 35 °C 200 rpm.

b Ratio determined by GC-FID analysis, average of three reactions. Products characterized by GC–MS and ^**1**^H NMR analyses. CFE = cell free extract.

### Enzyme Immobilization

The NR-55 and GDH-101 enzymes were not His-tagged, so use of affinity-based resins was not possible for immobilization.^34^ A recent report demonstrated that the NR from *Enterobacter cloacae* could be immobilized onto magnetic nanoparticles;^35^ however, this approach required synthesis of a specific Fe nanoparticle. Instead, we explored several commercially available supports from the Lifetech range, provided by Purolite, including ECR8285, ECR8309F, and ECR8204F. These contained a combination of epoxy (ECR8285 and ECR8204F) and amine functionalization (ECR8309F, which required pre-activation with glutaraldehyde). The NR-55 and GDH-101 enzymes were both successfully immobilized on all three resins (see the SI for details) and tested against **1a**. Under the optimum batch conditions developed in Table 1 (50 mM **1a**, 10% DMSO), all three reactions with the different supports went to completion in 2 h using immobilized enzymes. After leaving the resins in storage at 4 °C for 1 week, they were tested again under the same batch conditions. It was found that the mixture of NR-55 and GDH-101 immobilized on the ECR8309F resin still fully converted the material; however, the epoxy resins saw >50% loss in conversion. Therefore, the ECR8309F resins were taken forward for the flow experiments. As well as permitting transfer to continuous flow, our reuse experiments of the immobilized biocatalysts over the course of several days (see SI for details) demonstrates how immobilization can be used as a tool for increased efficiency, and improvement of the overall sustainability credentials of the reaction.

### Flow Experiments

With immobilized preparations in hand, our focus moved to the development of a continuous NR process. As stated, 2-chloro-5-nitropyridine **1a** was sparingly soluble in aqueous systems even with 10 wt % co-organic solvent mixtures. Therefore, optimization of the flow process proceeded at the maximum observed aqueous concentration of 7.5 mM **1a**. An Omnifit column was used to house the immobilized enzymes in a 4:1 ratio of NR-55:GDH-101, with both immobilized on ECR8309F. The final column contained 1 g of supported enzymes (10 wt % CFE) and delivered a column volume of 785 μL. Initially, a residence time (*t*_res_) of 10 min was set (flow rate = 79 μL min^–1^) and 68% conversion to **2a** was observed after four column volumes (GC-FID analysis), and the conversion quickly dropped off after this (Table 2, run 1). Optimization revealed it was in fact the 10% MeCN co-solvent that deactivated the enzyme after only a few hours of continuous contact time. This was not observed in batch reactions, perhaps due to the enzyme leaching from the support and remaining in solution, or the rate of the batch reactions meaning full conversion was reached prior to deactivation. Use of DMSO (10% v/v) ameliorated this issue and delivered longer-term stability of the enzyme. Under the same conditions, the reaction was run continuously for 36 column volumes (6 h) and full conversion of **1a** to **2a** was observed (Table 2, run 2). The same column was then used for a subsequent run under identical conditions 72 h later, with no observed deterioration in conversion (Table 2, run 3). To test the efficiency of the enzyme, the flow rate was doubled leading to half the *t*_res_ and, once again using the same column, the same volume was collected (36 column volumes, 3 h). This still led to full conversion throughout, demonstrating how well the immobilized enzyme performed (Table 2, run 4). The specific yield for the second column (runs 2–4) was calculated to be 5.88 g g_NR-55_^–1^ (the CFE had 10% protein by mass content, meaning approximately 8 mg of NR-55 protein was immobilized within the column). The isolated yield of each run was around 50% and was used to calculate the specific yield for this system. These experiments demonstrate the increased productivity that can be obtained with flow, through repeated reuse of the packed-bed reactor. However, the low aqueous solubility of **1a** also highlighted a limitation of using this system, with other nitroaromatics also likely to suffer solubility limits in buffer.

**Table 2:**
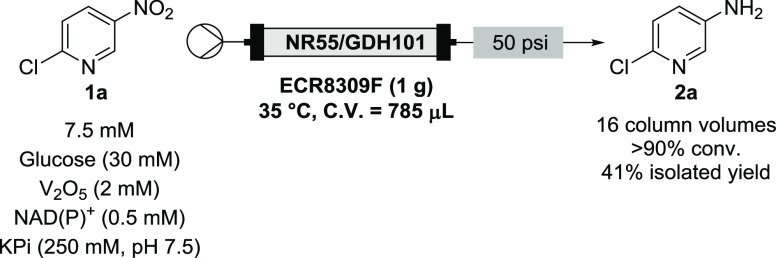
Optimization of Continuous NR-55 Reaction with **1a**

run	co-solvent (v/v)	*t*_res_	conv. (36 c.v.)	isolated yield (mass)	specific yield (g_**2a**_ g_NR-55_^–1^)^a^
1	10% MeCN	10 min	<5%		
2	10% DMSO	10 min	100%	52% (14 mg)	1.75^b^
3	10% DMSO	10 min	100%	48% (15 mg)	3.63^b^
4	10% DMSO	5 min	97%	53% (18 mg)	5.88^b^

a Based on Isolated Yield of **2a**; 8 mg of NR-55 protein calculated to be in the column.

b Accumulative as the same column was used.

Substrate solubility proved to be an overall limitation for this continuous NR-55 reaction, despite full conversion being obtained for **1a**. To ensure the productivity observed in batch with higher substrate concentrations could be replicated in flow, the hydrochloride salt of a related substrate, 3-nitro-4-methyl pyridine **1b**, was explored (attempts to form the hydrochloride salt of **1a** were unsuccessful in our hands). This could be dissolved to a concentration of 25 mM in the reaction solution. The reaction mixture was made with 100 mM glucose concentration and did not require DMSO as a co-solvent. A new column was prepared with freshly immobilized NR-55 and GDH-101 and the reaction run with a 10 min *t*_res_ due to the higher concentration of substrate **1b** (flow rate = 86 μL min^–1^). In total, 30 column volumes were collected to assess the longevity of the enzyme with regard to the higher substrate concentration (Table 3, run 1). These combined column volumes afforded 65 mg of a 2:1 conversion of desired product **2b** and starting material **1b**, with a trace of a dimerization product (azoxy or azo, unconfirmed) also observed by ^1^H NMR (see the SI). Pleasingly, doubling the *t*_res_ to 20 min (flow rate = 43 μL min^–1^) resulted in full conversion, which was maintained for 16 column volumes (Table 3, run 2). This was repeated with the same column on 3 consecutive days, with conversion being around 80% to the desired product by the end of the third run after eight column volumes (Table 3, runs 3 and 4). The collected fractions from all three reactions were pooled and extracted to afford 84 mg in a 21:1 mixture of **2b**:**1b**, equating to a 90% isolated yield and a specific yield of 10.6 g_**2b**_ g_NR-55_^–1^.

**Table 3:**
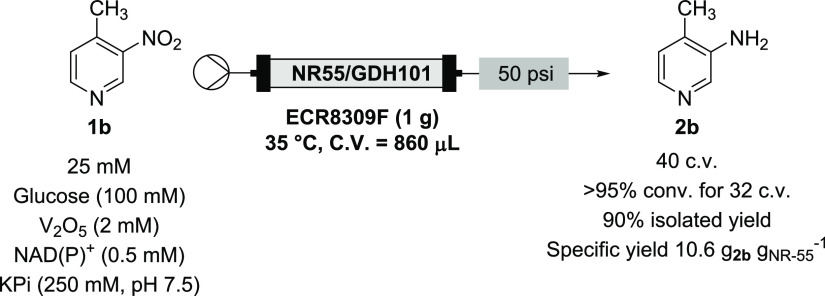
Optimization of Continuous NR-55 Reaction with **1b**

run	*t*_res_	conv. (8 c.v.)	conv. (16 c.v.)^a^
1	10 min	68%	50%
2	20 min	100%	100%
3	20 min	97% (14:1 **2b**:**1b**)	90% (8:1 **2b**:**1b**)
4	20 min^a^	81% (8:1 **2b**:**1b**)	n/a

a Based on isolated yield of **2b**; 8 mg of NR-55 protein calculated to be in the column. Trace of dimerization products also observed by ^1^H NMR analysis (see the Supporting Information).

**Scheme 2:**
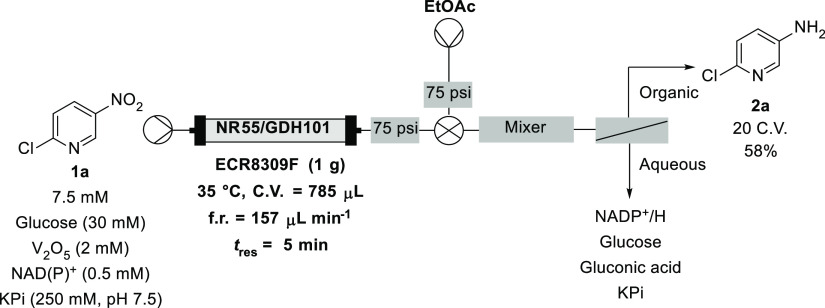
Continuous Extraction of Aniline Product, with an Isolated Yield of 58% (9 mg) Obtained through Evaporation of the Combined Fractions

One of the benefits of using continuous reactors is being able to incorporate methods to reduce processing steps. For example, using flow, continuous extraction has been demonstrated.^36^ Therefore, we investigated whether this could be applied to the continuous NR-55 reaction to improve the overall efficiency. A Zaiput membrane separator was therefore incorporated into the continuous setup in an attempt to enable this (Scheme 2). The Zaiput separator was preceded by 30 cm of 1/16″ ID tubing packed with sand to act as a static mixer, which connected via a T-junction to the aqueous stream from the biotransformation and a second pump, which delivered EtOAc. The nature of the piston pump required a BPR to be fitted before the flow to prevent pulsation and back flow of the aqueous phase into the EtOAc channel. The reaction was run for 20 column volumes, and analysis determined complete conversion to the desired product with full extraction to the organic phase, with no organic reaction components observed in the aqueous phase. Evaporation of the collected organic fractions followed by column chromatography afforded 9 mg of the desired product, which equated to a 58% isolated yield. This ability significantly reduced processing time and works particularly well with anilines as they are poorly soluble in aqueous phases compared with aliphatic amines, meaning no basification of the aqueous phase is necessary to enable full extraction. Low solubility of the starting material, as stated earlier, is likely the cause for the lower isolated yield, with precipitate formed in the starting material flask prior to entering the pumps expected to be **1a**.

**Scheme 3:**
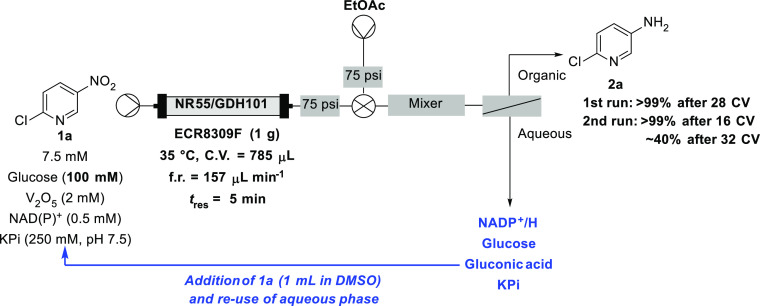
Recycling of the Aqueous Phase Permitted Implementation of a Closed-Loop Reactor

With this extraction process in hand, we speculated whether the separated aqueous phase could be reused as the organic material had been selectively removed and the gluconic acid produced was likely buffered by the high phosphate concentration. The reaction was run again for 28 column volumes, but with 100 mM glucose to start, and full conversion to **2a** was observed. Then, 18 mL of the aqueous phase was combined with a sample of pyridine **1a** in DMSO (2 mL, 75 mM) to afford a new reaction mixture with approximately the same concentration of **1a** as the initial reaction mixture. While some conversion was observed, it was low (<20% to **2a**). This may have been due to a higher concentration of DMSO having a negative impact on the enzymes. The first reaction was repeated (28 column volumes, >99% conversion), and then to the recycled aqueous phase (19 mL), a more concentrated solution of **1a** in DMSO (1 mL, 150 mM) was added to reduce the overall DMSO concentration (Scheme 3). Pleasingly, from a second run of the reaction using the recycled aqueous phase, full conversion to the desired amine **2a** was achieved with the recycled buffer solution for up to 16 additional column volumes. This then dropped steadily to only 40% after a further 16 column volumes but demonstrated this could be achieved with no additional nicotinamide being added to the reaction solution. The clear benefit of using this is the halving of the amount of both buffer and nicotinamide required. When calculating efficiency metrics, media make a significant contribution to the overall mass of a process.^37^ Therefore, re-using solvent will improve an overall process by significantly reducing the overall mass.

## Conclusions

We have shown that a nitroreductase enzyme (NR-55) is a highly active biocatalyst that is amenable to immobilization and transfer into a continuous reactor. The same packed-bed reactor column was reused on three days to deliver a specific yield of 5.88 g_1a_ g_NR-55_^–1^ for the synthesis of 5-amino-2-chloro-pyridine **2a**. The solubility of 2-chloro-5-nitropyridine **1a** limited the productivity of the system, so a hydrochloride salt of 3-nitro-4-methyl pyridine **1b** was used at higher concentrations and was able to achieve higher productivities of >10 g_**2b**_ g_NR-55_^–1^. The hydrophobic nature of the product anilines meant these reactions were amenable to continuous extraction without the need to basify aqueous streams. This allowed reclamation of the aqueous phase from the reaction and reuse of it, permitting a closed-loop reaction process to be established. This highly selective, low-energy method offers an alternative to the traditional chemical synthesis of aromatic amines, avoiding the high temperatures, expensive metal catalysts, and toxic acids, historically used. In particular, the immobilized enzymes used in this study have been previously shown to have a broad substrate scope,^18^ so the system described here has the potential to become a screening tool in the discovery of new aniline moieties. The immobilized preparations permit simple use in packed-bed reactors, as we have shown, and what this also allows is integration with other continuous operations. The continuous extraction unit is one example, but this could also be extended to subsequent packed-bed reactors with additional biocatalytic modules, or other synthetic transformations such as aniline substrates for continuous Buchwald–Hartwig transformations. Further work will focus on the wider development of immobilized NR complexes to permit use of organic solvents and expansion of the substrate scope.

## Supporting Information Available

Procedures and details for flow experiments, enzyme immobilization protocols, and representative ^1^H NMR and GC-FID data (PDF)
