# Supplementary material for: Realizing the Continuous Chemoenzymatic Synthesis of Anilines Using an Immobilized Nitroreductase
Source: ACS Sustain Chem Eng. 2023 Jun 2;11(23):8556–61. doi: 10.1021/acssuschemeng.3c01204 (PMC10265703; doi:10.1021/acssuschemeng.3c01204)
Supplement: Supplementary file 1 — sc3c01204_si_001.pdf [file sc3c01204_si_001.pdf]

Optimisation of an immobilised nitroreductase flow reaction – Supporting information

Sebastian C. Cosgrove,*[1] Gavin J. Miller,[1] Amin Bornadel,[2] & Beatriz Dominguez[2]

1. School of Chemical and Physical Sciences & Centre for Glycoscience Research and Training, Keele University, Keele, Staffordshire, ST5 5BG, United Kingdom.

2. Johnson Matthey, Cambridge Science Park, Milton Rd, Cambridge, CB4 0FP, United Kingdom.

Corresponding author email: s.cosgrove@keele.ac.uk; g.j.miller@keele.ac.uk

General experimental S2
Flow equipment S2
Enzyme immobilisation S2
Recovery and reuse experiment S3
Biotransformation using soluble biocatalysts S4
Biotransformation using immobilised biocatalysts S4
Flow reactions S4
^{1}H NMR Spectra S5
GC S9
References S9

General experimental

All reagents and solvents which were available commercially were purchased from Acros, Alfa Aesar, Fisher Scientific, Fluorochem, Sigma Aldrich or TCI. Biocatalysts were supplied in lyophilised cell-free extract form by Johnson Matthey, and were used without any additional processing. ^{1}H NMR spectra were recorded at 400 MHz using a Bruker Magnet system 400’54 Ascend. NMR data were analysed using MestReNova. ^{1}H NMR splitting patterns were assigned as follows: br. s (broad singlet), s (singlet), d (doublet), dd (doublet of doublets), ddd (doublet of doublet of doublets), app. t (apparent triplet), t (triplet), quartet (q) or m (multiplet and/or multiple resonances). Gas chromatography (GC) data was recorded using an Agilent 7820A GC-FID system. The column used was an Agilent HP-5 (30 m × 0.32 cm × 0.25 µm). The data was analysed using Agilent OpenLab software packages. The Lifetech ECR resins were supplied by Purolite (Llantrisant, UK). A Microfuge 20 tabletop centrifuge was purchased from Beckman Coulter life sciences (High Wycombe, UK). A Grant Bio ES-20 shaking incubator (up to 42 °C, 250 rpm) was purchased from Wolf labs.

Flow equipment

Continuous flow reactions were performed using the following equipment: Eldex Optos series pumps model 2SM (flow rate 0.003-5.000 mL min^{-1}) and PTFE tubing (I.D. 1/16”) were purchased from Cole Parmer (Cambridge, UK). An Azura P 4.1S pump (flow rate 0.001-10.000 mL min^{-1}) was purchased from Knauer (Germany). Omnifit adjustable glass columns (I.D. 6.6 mm, length 100 mm) were purchased from Cole Parmer (Cambridge, UK). Stainless steel and PEEK fittings were purchased from Swagelok (Manchester, UK). A Gilson FC203 110/220V fraction collector was purchased from Gilson (Dunstable, UK). An SSI flow-through back-pressure regulator (5-75 psi) was purchased from Sigma-Aldrich (Gillingham, UK). Omnifit manual three-way switching valves were purchased from Fisher scientific UK. Kinesis microstatic mixers (for 1/16” tubing) were purchased from Fisher scientific UK. A Zaiput SEP-10 liquid-liquid (flow rate up to 10 mL min^{-1}) separator was purchased from Zaiput Flow Technologies.

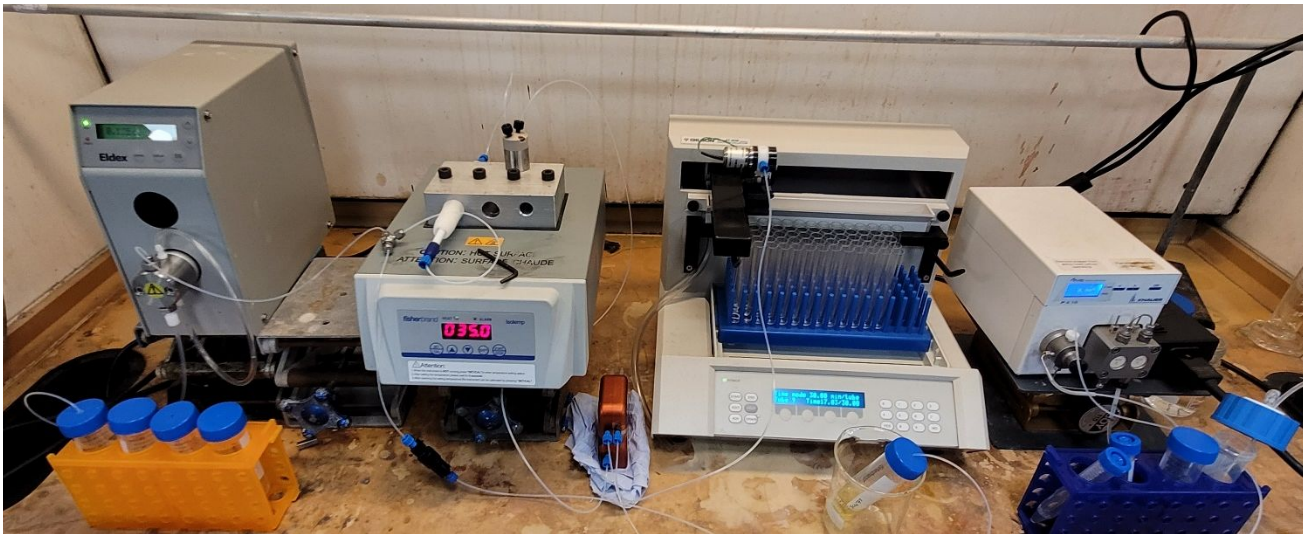

Enzyme immobilisation

The NR-55 and GDH-101 biocatalysts were immobilised onto the Lifetech ECR amino-functionalised (ECR8039F) and epoxy-functionalised (ECR8285 and ECR8304) resins according to the suppliers guidelines. The properties of the resins are shown in table S1.

Resin	Functionality	Size (µm)	Pore diameter (Å)
ECR8309F	Amino	300-710	600-1200

ECR8204F	Epoxy	150-300	300 – 600
ECR8285	Epoxy butyl	250-1000	450 – 650

Table S1. Properties of Lifetech ECR resins

Modified procedures were followed according to the functionality of the resin:

Amino: The amino resin (900 mg per 100 mg of lyophilised CFE) was washed three times with immobilisation buffer (20 mM KPi, pH 7.5; 4 mL per 900 mg of resin). After, a 2% glutaraldehyde solution in immobilisation buffer was added to the resin (4 mL per 900 mg of resin) and the resin was placed on an orbital shaker at 18 rpm for one hour. The resin was then washed three times with immobilisation buffer (4 mL per 900 mg of resin). The lyophilised CFE was then dissolved in immobilisation buffer (100 mg per 4 mL) and added to the activated resin. This was placed an orbital shaker at 18 rpm for 20 hours. The resin was then washed four times with post-immobilisation solution (20 mM KPi, 250 mM NaCl, pH 7.5, 10 mL) and the resin stored at 4 °C until required.

Epoxy: The epoxy resin (900 mg per 100 mg of lyophilised CFE) was washed three times with immobilisation buffer (20 mM KPi, pH 7.5; 4 mL per 900 mg of resin). The lyophilised CFE was then dissolved in immobilisation buffer (100 mg per 4 mL) and added to the washed resin. This was placed an overhead rotator at 18 rpm for 20 hours. This was left to stand for 24 hours at room temperature. The resin was then washed four times with post-immobilisation buffer (20 mM KPi, 250 mM NaCl, pH 7.5, 10 mL) and the resin stored at 4 °C until required.

Recovery and reuse experiment

A sample of the ECR8309F resin was washed, stored at 4 °C and used again 24 hours later (Figure S1, day 8 after immobilisation). It was still able to produce **2a**, with >90% conversion (Figure S1, day 9), and after recovery and storage for three more days at 4 °C could still obtain >75% conversion to **2a** under the same conditions (Figure S1, day 12). One more recovery and re-use of the same sample showed conversion around 60% to **2a** (Figure S1, day 13). This demonstrated that the ECR8309F immobilised NR-55 could be re-used for up to 12 days, before losing significant activity, aligning well to the needs of a continuous system. Lyophilisation of the immobilised NR-55 samples resulted in apparent loss of the flavin co-factor after re-hydration, so was not suitable for long-term storage of the enzyme. The GDH-101-ECR8309F could be stored long term in the fridge (three months) without being compromised.

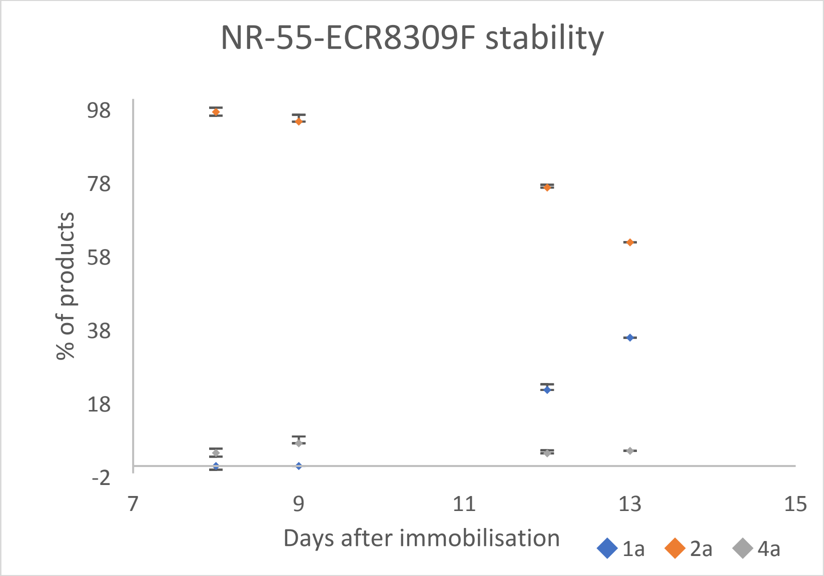

Figure S1. Reusability of the immobilised NR-55ECR8309F material for reduction of nitropyridine **1a**. Conditions: Substrate **1a** (50 mM), NR-55ECR8309F (50 mg mL^{-1}), GDH-101ECR8309F (10 mg mL^{-1}), Glucose (200 mM), $NADP^+$ (1 mM), V_2O_5 (2 mM), DMSO (10% v/v), 35 °C, 200 rpm. Conversion determined by GC-FID analysis, average of three reactions.

Biotransformation using soluble biocatalysts

In an Eppendorf tube (volume = 1.5 mL, final reaction volume = 500 µL), was added KPi buffer (315 µL, 250 mM, pH = 7.5), glucose (50 µL, final conc. = 200 mM, stock solution = 2 M), $NADP^+$ (5 µL, final conc. = 1 mM, stock solution = 100 mM), V_2O_5 (50 µL, final conc. = 2 mM, stock solution = 20 mM), NR-55 (50 µL, final conc. = 5 mg mL^{-1}, stock solution = 50 mg mL^{-1}), GDH-101 (50 µL, final conc. = 1 mg mL^{-1}, stock solution = 10 mg mL^{-1}), substrate (50 µL, final conc. = 50 mM, stock solution = 500 mM [in organic solvent]). This was incubated at 35 °C with 200 rpm shaking for 2 hours. After, 5 M NaOH (20 µL) and EtOAc (1 mL) were added, and the solutions were vortexed for 5 seconds. These were centrifuged 2 minutes at 14,000 rpm. The organic phase was then decanted and analysed by ^1H NMR or GC-FID.

Biotransformation using immobilised biocatalysts

In an Eppendorf tube (volume = 1.5 mL, final reaction volume = 500 µL), was added immobilised NR-55 on ECR8309F (50 mg, final conc. = 100 mg mL^{-1}) and immobilised GDH-101 on ECR 8309F (5 mg, final conc. = 10 mg mL^{-1}). To this was added KPi buffer (415 µL, 250 mM, pH = 7.5), glucose (50 µL, final conc. = 200 mM, stock solution = 2 M), $NADP^+$ (5 µL, final conc. = 1 mM, stock solution = 100 mM), V_2O_5 (50 µL, final conc. = 2 mM, stock solution = 20 mM), and substrate (50 µL, final conc. = 50 mM, stock solution = 500 mM [in organic solvent]). This was incubated at 35 °C with 200 rpm shaking for 2 hours. After, these were centrifuged 2 minutes at 14,000 rpm. The supernatant was collected and analysed as required. The resin was either discarded or washed with buffer and stored at 4 °C to allow reuse.

Flow reactions

The immobilised enzymes were combined to uniform heterogeneity in a suspension in buffer, and were decanted into the Omnifit column. The column was sealed and then adjusted to remove any air from the internal cavity in the column. When column was packed and sealed, the cavity volume was calculated according to the manufacturer's guidelines (0.3421 x bed length in cm, i.e., 2.2 cm x 0.3421 = 0.752 mL internal volume).

Prior to use, the pump was washed with d.H_2O (F.R. = 2 mL min^{-1} for 10 minutes) to ensure removal of any residual isopropyl alcohol in the pump head and tubing. The tubing was then connected to the reactor column, which was secured in the dry bath heating block which was set to 35 °C. The heating block was switched on at least 30 minutes prior to use to ensure uniform heating. The system was then primed with reaction buffer (F.R. = 0.5 mL min^{-1} for 10 minutes). Ensuring there were no leaks in the system, the flow rate was then set to the required speed to enable the appropriate residence time. The reaction was run for the required amount of time, with fractions collected using a fraction collector. When the reaction was completed, the reaction mixture was analysed and processed. The system was then flushed with KPi buffer (F.R. = 0.5 mL min^{-1} for 10 minutes) and the effluent discarded. The column was removed and stored at 4 °C until required. The pump and tubing were washed with 80% EtOH or isopropyl alcohol (F.R. = 2 mL min^{-1} for 10 minutes) and left until next required.

^{1}H NMR Spectra

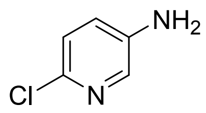

2-chloro-5-amino pyridine 2a: δ H (400 MHz, $CDCl_3$) 7.85 (1H, d, *J* = 3.0, H-6), 7.08 (1H, d, *J* = 8.5, H-3), 6.96 (1H, dd, *J* = 8.5, 3.0, H-4). The data is in accordance with the literature.[1]

^{1}H NMR Spectra **2a**

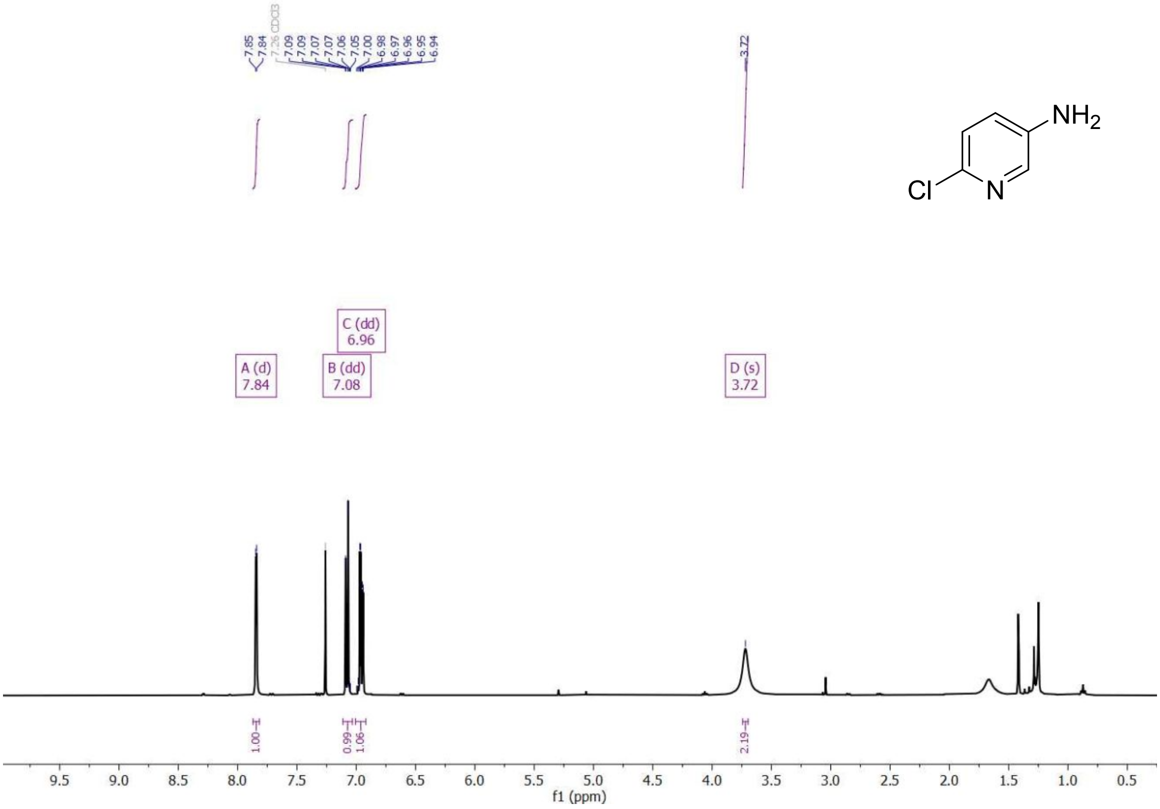

^{1}H NMR spectra of **1a** products of reduction (**2a**, **3a** and **4a**)

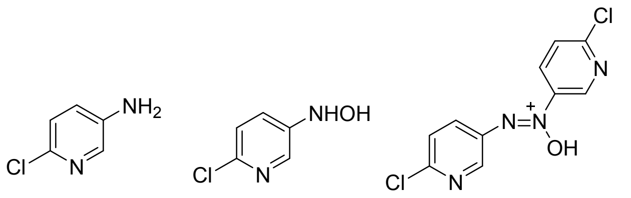

2a **3a** **4a**

3a: δ H (400 MHz, $CDCl_3$) 8.11 (1H, d, *J* = 2.9, H-6), 7.32 (1H, dd, *J* = 8.6, 2.9, H-4), 7.21 (1H, d, *J* = 8.6, H-3).
4a (aromatic systems not defined): δ H (400 MHz, $CDCl_3$) 9.33 (1H, d, *J* = 2.8, H-6’), 9.08 (1H, d, *J* = 2.6, H-6), 8.71 (1H, dd, *J* = 8.7, 2.6, H-4’), 8.55 (1H, dd, *J* = 8.7, 2.8, H-4), 7.52 (1H, d, *J* = 8.7, H-3’), 7.47 (1H, d, *J* = 8.7, H-3).

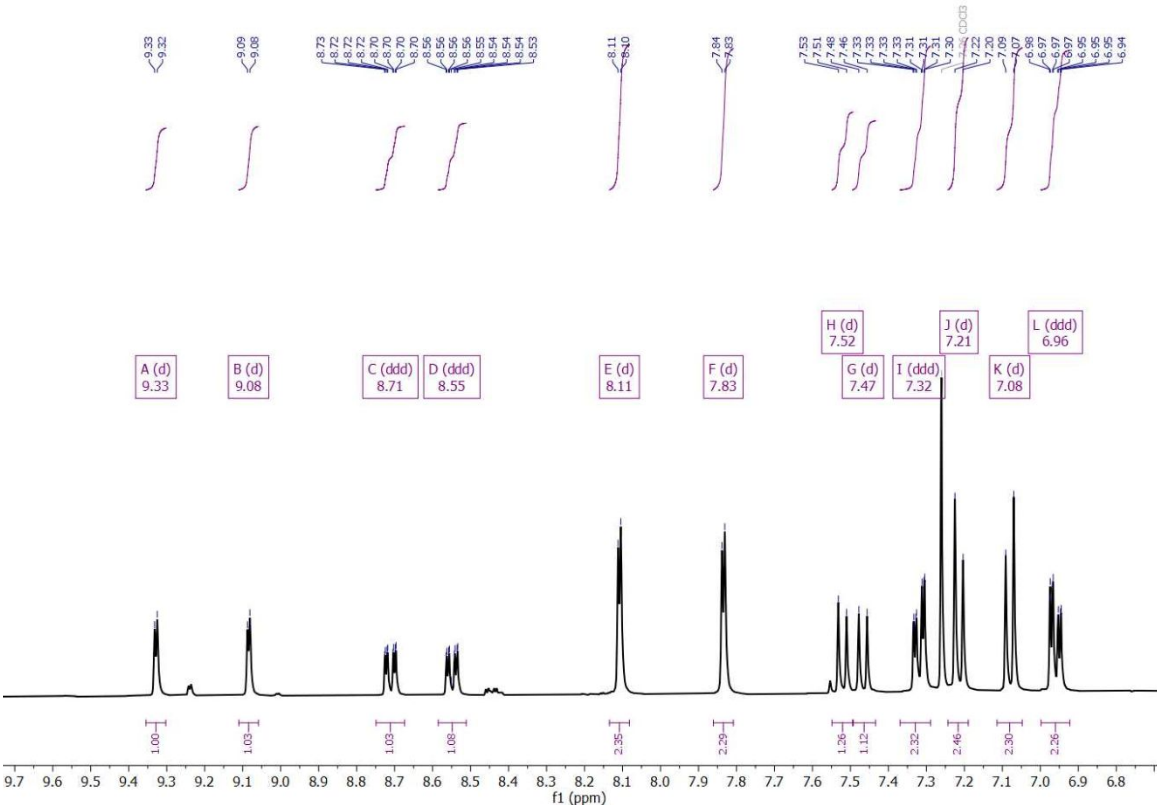

Timecourse experiment showing conversion at 30 minutes (red), 1 hour (green) and 2 hours (blue) for NR-55 reduction of **1a**.

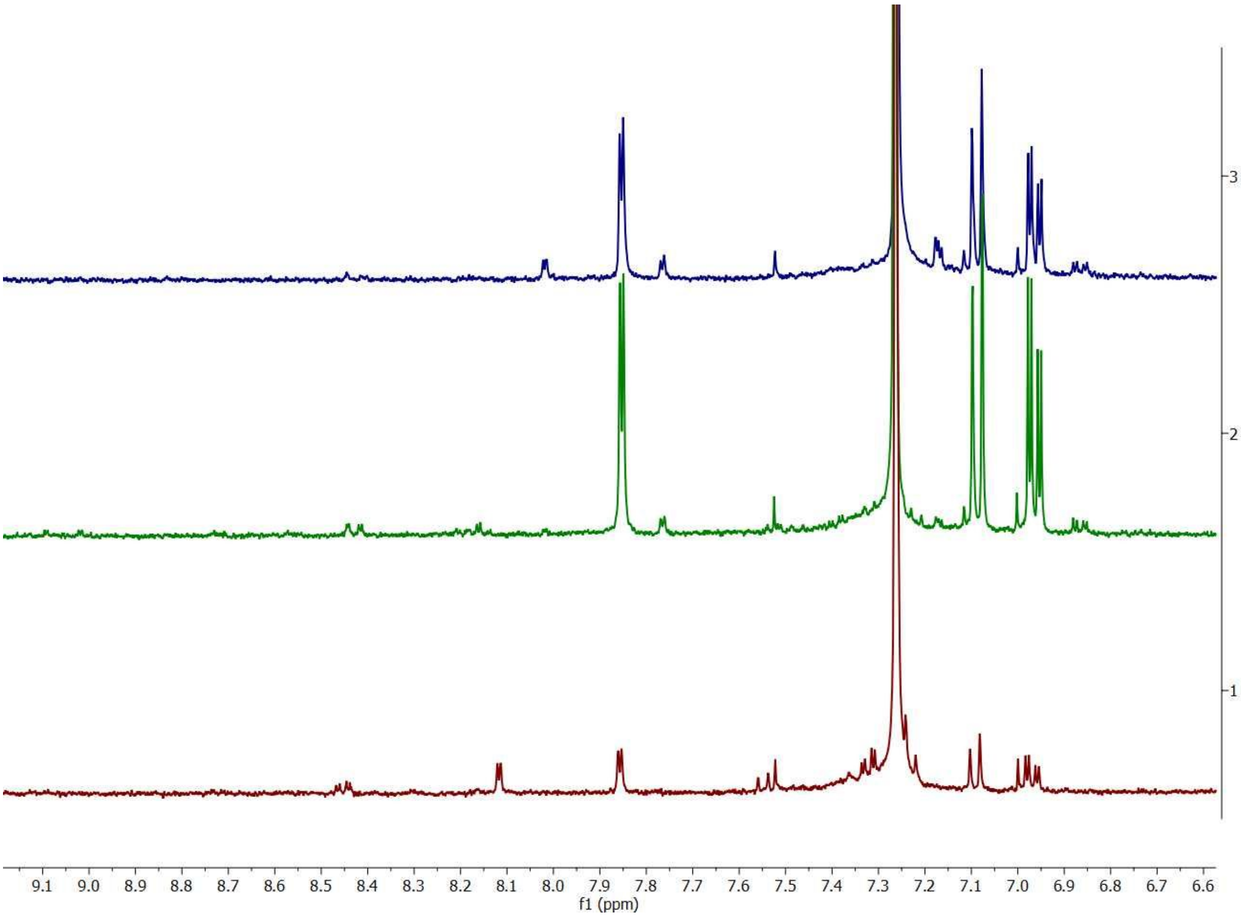

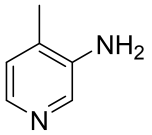

3-amino-4-methyl pyridine 2b: δ H (400 MHz, $CDCl_3$) 8.02 (1H, s, H-2), 7.94 (1H, d, *J* = 4.8, H-5), 1.95 (1H, d, *J* = 4.8, H-6), 3.59 (2H, br. s, NH_2).[2]

^{1}H NMR Spectra **2b**

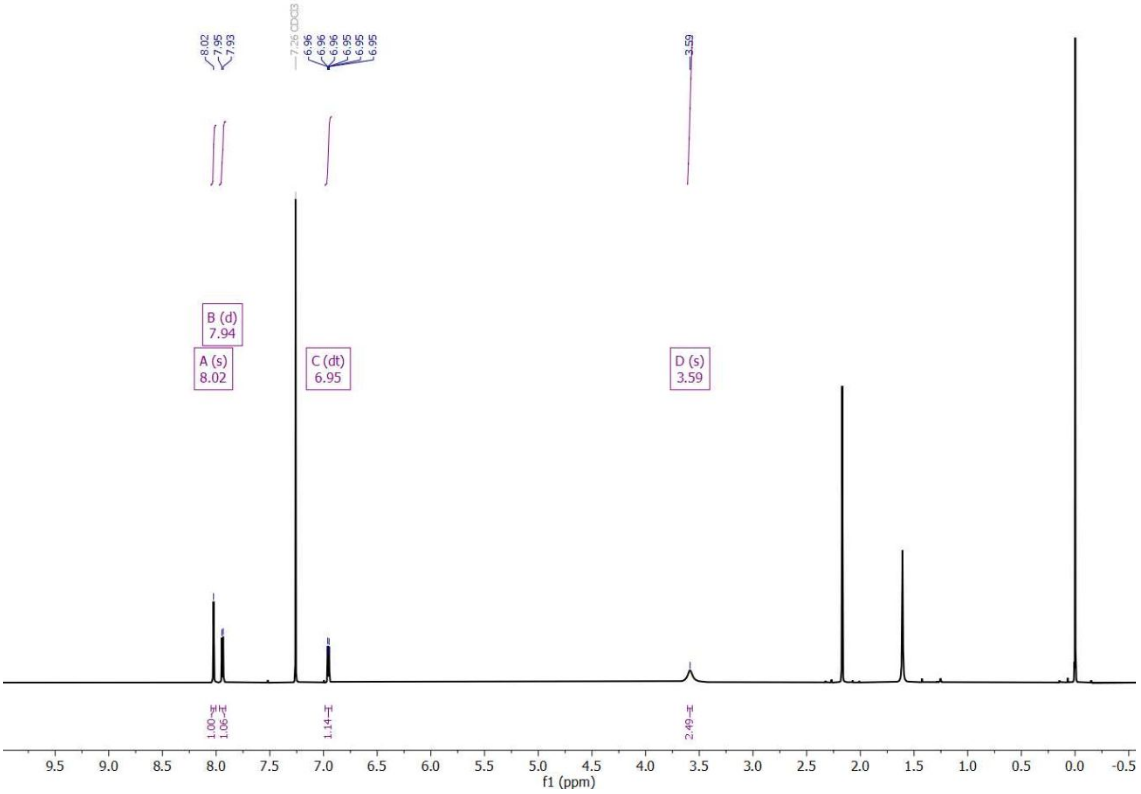

^{1}H NMR spectra of **1b** products of reduction (**1b**, **2b**, and **4b**)

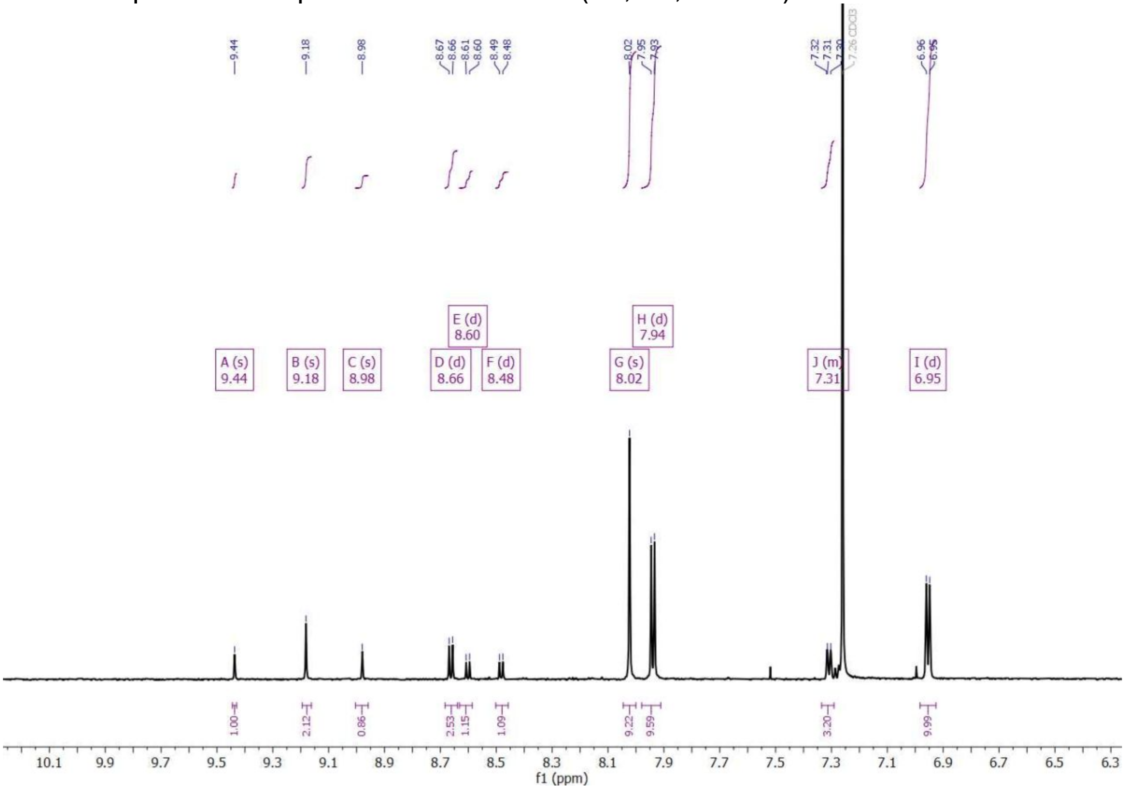

GC

Method: 1.2 mL min^{-1} , 100 °C hold for 1 minute, 100 °C to 300 °C at 10 °C min^{-1}, 300 °C hold for 2 minutes, injector 250 °C, detector 300 °C.

Traces showing **1a** (retention = 4.908 min) and **2a** (retention = 5.917 min) after A) first run of a flow reaction and then B) after recycling of aqueous phase.

A)

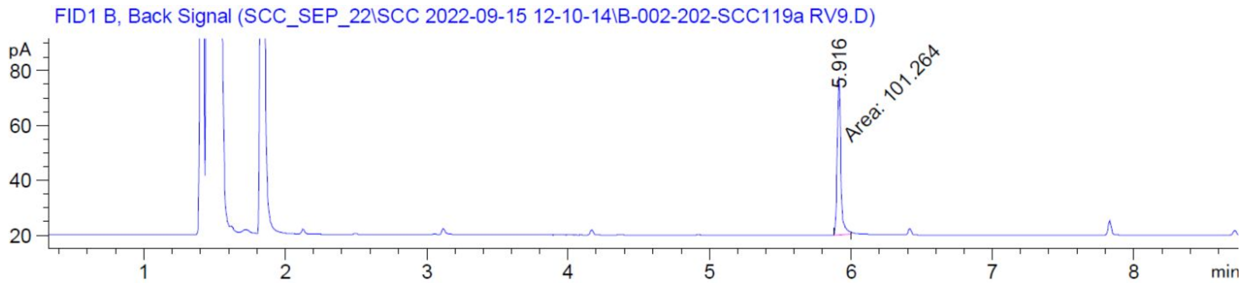

B)

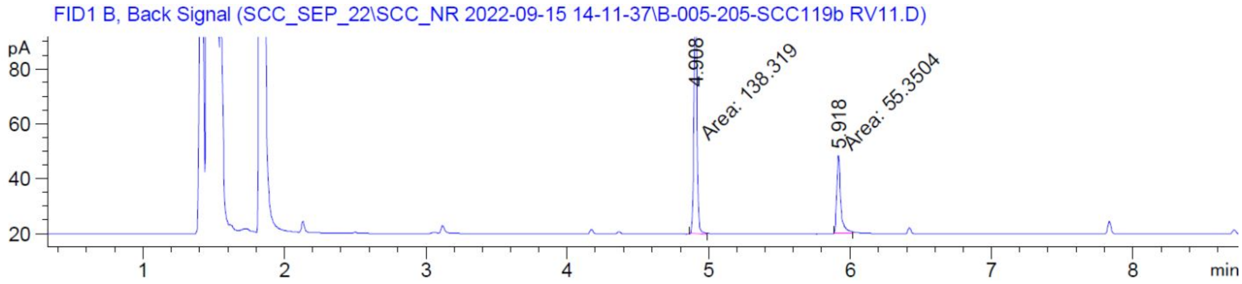

References

1 H. C. Du, N. Simmons, J. C. Faver, Z. Yu, M. Palaniappan, K. Riehle and M. M. Matzuk, *Org. Lett.*, 2019, **21**, 2194–2199.

2 F. Du, Q. Zhou, Y. Fu, Y. Chen, Y. Wu and G. Chen, *Synlett*, 2019, **30**, 2161–2168.
